# Change in cardiovascular health and rate of cognitive decline in older adults: a 15-year population-based study

**DOI:** 10.1186/s12877-024-04856-y

**Published:** 2024-03-18

**Authors:** Andreja Speh, Milica G. Kramberger, Bengt Winblad, Lars Bäckman, Chengxuan Qiu, Erika J. Laukka

**Affiliations:** 1 Aging Research Center, Department of Neurobiology, Care Sciences and Society, Karolinska Institutet- Stockholm University, Tomtebodavägen 18A, Solna, Stockholm, Sweden; 2 Division of Neurogeriatrics, Department of Neurobiology, Care Sciences and Society, Karolinska Institutet, Stockholm, Sweden; 3 Department of Neurology, University Medical Center Ljubljana, Ljubljana, Slovenia; 4 Medical Faculty, University of Ljubljana, Ljubljana, Slovenia; 5 Theme Inflammation and Aging, Karolinska University Hospital, Huddinge, Sweden; 6 Stockholm Gerontology Research Center, Stockholm, Sweden

**Keywords:** Aging, Cardiovascular risk factors, Cognition, Epidemiology

## Abstract

**Background:**

Previous research on associations between cardiovascular health, measured at a single timepoint, and rate of age-related cognitive decline shows divergent findings dependent on the participants’ age and the health metric studied. The aim of this study was to add to the knowledge in this field by investigating whether change in cardiovascular health, assessed with Life’s Simple 7 (LS7) score, is associated with rate of cognitive change in young-old and old-old adults.

**Methods:**

The study included 1022 participants aged ≥ 60 years from the Swedish National Study on Aging and Care-Kungsholmen (SNAC-K), who underwent repeated neuropsychological testing (episodic memory, semantic memory, verbal fluency, and perceptual speed) across up to 15 years. LS7, composed of seven cardiovascular health metrics (smoking, diet, physical activity, body mass index, plasma glucose, total serum cholesterol, and blood pressure), was assessed at baseline and at the 6-year follow-up. Change in LS7 was calculated as the difference between baseline and 6 years (range − 5 to 8 points) and categorised into worse (−5 to −2 points), stable (−1 to 1 points), and improved (2 to 8 points). Change in cognitive performance as a function of LS7 change categories was estimated using linear mixed-effects models.

**Results:**

Participants were classified as stable (67.1%), improved (21.0%), or worse (11.8%) according to changes in LS7 score. Both the worse and improved categories were associated with faster cognitive decline. Age-stratified analyses revealed that worsening of LS7 was clearly associated with faster cognitive decline in the old-old (≥ 78 years), whereas improvement tended be associated with faster cognitive decline in the young-old (< 78 years) group.

**Conclusions:**

Change in cardiovascular health in old age may lead to accelerated cognitive decline, particularly in late senescence. These results suggest that it is important to monitor and maintain cardiovascular health status in very old adults.

**Supplementary Information:**

The online version contains supplementary material available at 10.1186/s12877-024-04856-y.

## Introduction

Vascular risk factors (VRF) have been associated with increased risk of dementia and an accelerated age-related cognitive decline [1]. However, some discrepant results have been reported and different patterns have been identified depending on the specific risk factor examined and the persons’ age [2]. For example, Liang et al. (2020) [3] observed different patterns of associations between VRF and dementia risk depending on whether they were assessed in mid-life or late-life, with ideal cardiovascular health metrics assessed at midlife being associated with lower risk of dementia. Another study found that while late-life cardiovascular risk factors were associated with cognitive declines in persons below 80 years, they were linked to gains in old-old age [4]. Our previous research, using the same population-based cohort as the present study, uncovered diverging patterns in the association between VRF and cognitive decline among young-old (< 78 years) and old-old (≥ 78 years) individuals [5]. We found that poor cardiovascular health was associated with faster cognitive decline only in young-old participants. This aligns with previous research suggesting that ideal cardiovascular health (e.g. low blood pressure and low body mass index (BMI)) might reflect a negative change in health status in very old adults [6, 7].

Life’s Simple 7 (LS7), proposed by the American Heart Association (AHA), is a measure of optimal cardiovascular health, composed of seven modifiable risk factors: smoking, diet, physical activity, BMI, plasma glucose, total serum cholesterol, and blood pressure [8]. Previous studies have demonstrated that a higher LS7 score is associated with better cognitive function [9, 10] and slower rate of cognitive decline in older adults [11, 12]. However, very few studies have investigated the relationship between changes in LS7 or other scores of multiple VRF and rate of cognitive change. Such studies might help explain discrepancies observed in previous research, based on cross-sectional cardiovascular health status, and give important clues regarding potential effects of large-scale interventions targeting cardiovascular health in different populations. One study from Betula examined how the trajectory of cardiovascular risk affects subsequent dementia risk and episodic memory decline over a period of 25 years. An accelerated cardiovascular risk, compared to stable risk, predicted an increased likelihood of developing dementia, and was further associated with an increased risk of memory decline [13]. Relatedly, in a 2-year intervention trial, a reduction in VRF was linked to less decline in hippocampal volume in the intervention group [14]. Furthermore, change in metabolic syndrome, especially fasting glucose and blood pressure, has been associated with dementia risk and cognitive performance [15, 16].

Studies that have focused on change in individual VRF and their associations with cognitive decline have produced mixed results. For example, longitudinal relations of blood pressure to cognition are predominantly nonlinear and moderated by age [17]. In persons aged 65 to 74 years, higher baseline blood pressure has been associated with worse cognitive function 11 years later, while in older age (≥ 75 years), higher blood pressure seemed to be related to better cognitive function at the end of follow-up [18]. For cholesterol, high levels during late life have been associated with decreased risk of dementia and slower cognitive decline [19, 20].

In sum, many studies examining the association between VRF and cognition in older adults have been limited by measuring individual VRF or by assessing cardiovascular health at a single time point. To gain a better understanding of the relationship between VRF and change in cognitive performance, it is important to track VRF over time. The aim of our study was to address this gap in the existing literature by examining how changes in individual and composite cardiovascular health metrics are associated with cognitive decline in different domains in old age. For this, a longitudinal population-based sample of young-old (< 78 years) and old-old (≥ 78 years) adults with a repeated assessment of LS7 and cognition across 6 and 15 years, respectively, was used.

## Methods

### Participants

Participants were derived from the population-based Swedish National Study on Aging and Care-Kungsholmen (SNAC-K, http://www.snac-k.se). The baseline sample included 3363 individuals belonging to pre-specified age cohorts of 60, 66, 72, 78, 81, 84, 87, 90, 93, 96, and 99 years and older. The participants are re-examined each time they reach the age of the next cohort (i.e., every 6 or 3 years). Each examination consists of a nurse interview, a medical examination, and a neuropsychological testing session. Summary of variables measured in SNAC-K is displayed in Supplementary Table 1. Participants with a diagnosis of dementia (DSM IV criteria, *n* = 321), or Parkinson’s disease (CERAD criteria, *n* = 24), schizophrenia (*n* = 13), developmental disorder (*n* = 3), history of stroke (*n* = 165), missing cognitive data (*n* = 281), or missing data on cardiovascular score at baseline (*n* = 718) were excluded. Because the focus of this study was on changes in LS7, we further excluded those with missing LS7 data at the 6-year follow-up (*n* = 816). Compared to participants who had data for LS7 at 6 years, those with missing data were older, more likely to be women, less educated, and have lower MMSE and LS7 total score at baseline (*p* < 0.001). The final sample included 1022 individuals. Of these, twenty-four individuals had missing cognitive data at follow-up. Those aged 60 to 72 years at baseline (M = 64.7, SD = 4.6, *n* = 864) were categorised into a young-old group and those 78 years or older (M = 80.6, SD = 3.0, *n* = 158) into an old-old group. Due to the study design, there were no individuals aged 73 to 77 years at baseline.

### Assessment of cardiovascular health

A detailed description of data collection, the definitions, and the categorisation of LS7 score in SNAC-K has been provided previously [5]. LS7 was calculated for baseline and for the 6-year follow-up, following the same procedure. Smoking habits were assessed during the nurse interview. Diet and physical activity were assessed with questionnaires. BMI was calculated as weight (kg) divided by squared height (m). For plasma glucose, glycated haemoglobin (HbA1c) level was measured, with a 1.1% adjustment for international values. Diabetes was recorded on the basis of self-reported medical history, hypoglycaemic drug use, diagnosis in the Swedish National Patient Register (ICD-10 code E11), or HbA1c ≥ 6.5% (48 mmol/mol). Total serum cholesterol was initially measured non-fasting; if ≥ 6.5 mmol/l, fasting total cholesterol was also measured and the mean of both was used. Arterial blood pressure was measured twice at a 5-min interval on the left arm in a sitting position using a sphygmomanometer and the mean of the two readings was used.

We classified all medications according to the Anatomical Therapeutic Chemical (ATC) classification system. Current use of cholesterol-lowering (ATC code C10) and antihypertensive (ATC codes C02, C03, C07, C08, and C09) drugs was assessed for baseline and follow-up.

For each metric on LS7, participants were categorised into a poor (score, 0), intermediate (1), or optimal (2) cardiovascular health group (see Table 1). We followed the cut-off values as applied by Sabia et al. [21] with some minor modifications for the glucose metric [22]. The sum of all seven metrics was used to calculate the total score, ranging from 0 to 14. Change in LS7 was computed as difference between baseline and 6-year score, and ranged from − 5 to 8 points. Individuals were categorised into a worse (−5 to −2 points), stable (−1 to 1 points), and improved (2 to 8 points) LS7 group.

**Table 1:** Definitions of Life Simple 7 score metrics (adapted from Sabia et al. [21])

Metrics	Poor (score, 0)	Intermediate (score, 1)	Optimal (score, 2)
Smoking	Current smoker	Stopped in the last 5 years	Never smoked or stopped > 5 year ago
Diet	Consumption of fruit and vegetables < 2 times per day AND no consumption of high fibre bread	Consumption of fruit and vegetables ≥ 2 times per day OR consumption of high fibre bread	Consumption of fruit and vegetables ≥ 2 times per day AND consumption of high fibre bread
Physical activity	Never, < 2–3 times per month, 2–3 times per month in light and/or moderate/intense exercise	Light exercise several times per week or every day	Moderate/intense exercise several times per week or every day
Body mass index	≥ 30 kg/m^2^	25−29.9 kg/m^2^	< 25 kg/m^2^
Plasma glucose	HbA1c ≥ 6.5% OR self-reported medical history, hypoglycaemic drug use, or diagnosis in the NPR	HbA1c 5.7–6.5% and no diabetes	Hb1Ac < 5.7%
Total serum cholesterol	≥ 240 mg/dl	< 200 mg/dl treated OR 200–239 md/dl	< 200 mg/dl untreated
Systolic and diastolic blood pressure	SBP ≥ 140 mm Hg OR DBP ≥ 90 mm Hg	SBP < 120 mm Hg and DBP < 80 mm Hg treated OR SBP 120–139 OR DBP 80–89 mm Hg	SBP < 120 mm Hg and DBP < 80 mm Hg untreated

HbA1c = glycated hemoglobin, SBP = systolic blood pressure, DBP = diastolic blood pressure

### Assessment of cognitive function

Cognitive performance was assessed at each time point according to a standardised procedure [23]. Episodic memory was assessed with free recall and word recognition [24]. For semantic memory, a 30-item vocabulary task was used [25]. Verbal fluency was assessed with both letter and category fluency [26]. For perceptual speed, digit cancellation [27] and pattern comparison [28] tests were used.

The cognitive test scores were z-transformed according to their baseline mean and standard deviation. The standardised scores were used to create composite scores for each cognitive domain by calculating a mean score when more than one test was available. For participants with data on at least two domains, a global cognition score was created, taking the mean of all available scores.

### Statistical analyses

Group differences were determined using chi-square tests for dichotomous variables and ANOVAs for continuous variables. Multinomial logistic regression was performed to evaluate whether LS7 baseline category (poor, intermediate, optimal) predicted LS7 change category change (worse, stable, improved), controlling for age at baseline, sex, and years of education. The association between categories of LS7 change and rate of change (slope) in different cognitive domains across 15 years was estimated using linear mixed-effect models. Follow-up time in years from baseline was used as the time scale. Fixed effects included LS7 change (worse, stable, and improved), time in study, and an interaction term for the grouping variable and time. Random intercept and slope were estimated as the random part of the mixed-effects model. Unstructured variance-covariance matrices were used for all models. All analyses were controlled for age at baseline, sex, years of education, and baseline LS7, centred at the sample mean.

For the outcome global cognition, the conventional α-level of 0.05 was used to signify statistical significance. For the four specific cognitive domains, a more conservative approach was adopted. A Bonferroni correction was implemented to account for multiple comparisons yielding a revised P-value of 0.0125 (0.05/4) as the threshold for statistical significance.

## Results

**Fig. 1:**
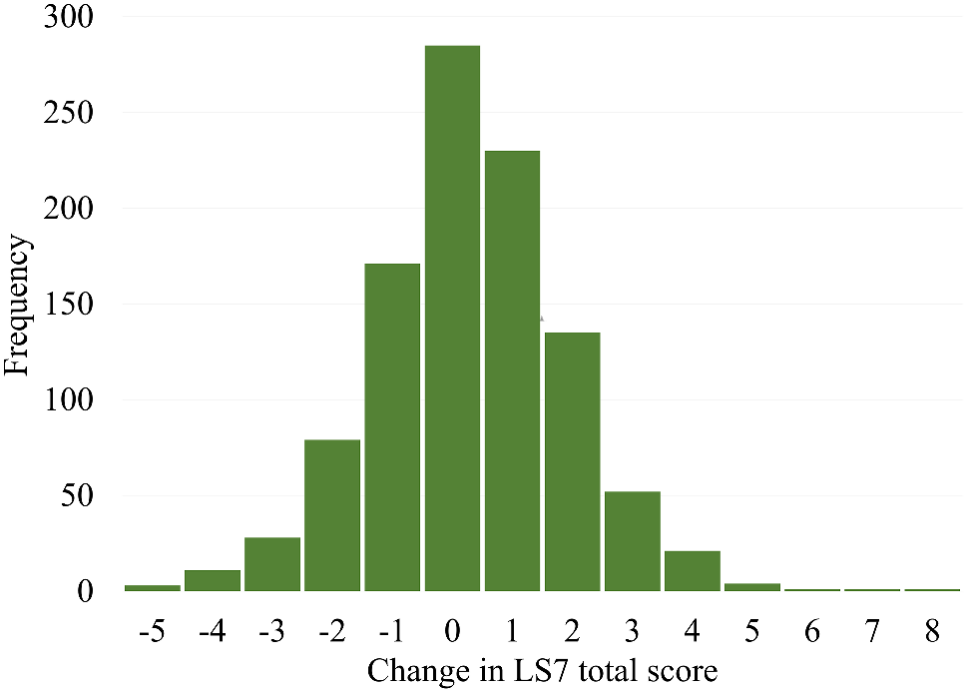
Distribution of change in LS7 total score from baseline to 6-year follow-up (*n* = 1022)

As shown in Fig. 1, change in LS7 score was normally distributed and ranged from − 5 to 8 points. On average, participants slightly improved (M = 0.3, SD = 1.6) their cardiovascular health from baseline to 6-year follow-up. Most of the participants were categorised as stable (*n* = 686, 67.1%), followed by improved (*n* = 215, 21.0%) and worse (*n* = 121, 11.8%). Baseline characteristics of the total sample and two age groups split by LS7 change are displayed in Table 2. There were no significant differences in sex, age, or education for the LS7 change categories.

**Table 2:** Descriptive statistics of study participants by categories of LS7 change

	Worse	Stable	Improved	p value
**Total sample (n = 1022)**				
N (%)	121 (11.8)	686 (67.1)	215 (21.0)	
Age, M (SD)	66.6 (7.0)	67.4 (7.2)	66.9 (7.5)	0.507
Female, N (%)	74 (61.2)	411 (59.9)	133 (61.9)	0.866
Years of education, M (SD)	13.5 (4.0)	13.2 (4.1)	13.4 (3.9)	0.591
MMSE score, M (SD)	29.5 (0.8)	29.3 (0.8)	29.4 (0.8)	0.192
*APOE* E4 carrier, N (%)	29 (24.0)	201 (29.3)	68 (31.8)	0.335
CVD, N (%)	13 (10.7)	76 (11.1)	19 (8.8)	0.646
LS7, M (SD)	9.3 (1.8)	7.8 (1.7)	6.5 (1.7)	< 0.001
BMI poor, N (%)	10 (8.3)	91 (13.3)	38 (17.7)	0.005
smoking poor, N (%)	13 (10.7)	77 (11.2)	50 (23.3)	< 0.001
physical activity poor, N (%)	3 (2.5)	85 (12.3)	47 (21.9)	< 0.001
cholesterol poor, N (%)	34 (28.1)	273 (39.8)	129 (60.0)	< 0.001
glucose poor, N (%)	5 (4.1)	39 (5.7)	14 (6.5)	0.022
blood pressure poor, N (%)	39 (32.2)	436 (63.6)	153 (71.2)	< 0.001
diet poor, N (%)	15 (12.4)	170 (24.8)	105 (48.8)	< 0.001
new antihypertensive medication at FU, N (%)	19 (15.7)	134 (19.5)	41 (19.1)	0.612
new anticholesterol medication at FU, N (%)	11 (9.1)	84 (12.2)	48 (22.3)	< 0.001
**Young-old (n = 864)**				
N (%)	103 (11.9)	579 (67.0)	182 (21.1)	
Age, M (SD)	64.3 (4.5)	65.0 (4.7)	64.3 (4.5)	0.143
Female, N (%)	64 (62.1)	340 (58.7)	114 (62.6)	0.572
Years of education, M (SD)	13.4 (4.1)	13.4 (4.0)	13.6 (3.9)	0.92
MMSE score, M (SD)	29.5 (0.8)	29.4 (0.8)	29.5 (0.7)	0.476
*APOE* E4 carrier, N (%)	26 (25.2)	172 (29.7)	60 (33.1)	0.402
CVD, N (%)	10 (9.7)	56 (9.7)	10 (5.5)	0.209
LS7, M (SD)	9.2 (1.8)	7.9 (1.7)	6.5 (1.8)	< 0.001
BMI poor, N (%)	9 (8.7)	77 (13.3)	30 (16.5)	0.038
smoking poor, N (%)	13 (12.6)	71 (12.3)	49 (26.9)	< 0.001
physical activity poor, N (%)	3 (2.9)	73 (12.6)	41 (22.5)	< 0.001
cholesterol poor, N (%)	29 (28.2)	232 (40.1)	109 (59.9)	< 0.001
glucose poor, N (%)	4 (3.9)	29 (5.0)	13 (7.1)	0.064
blood pressure poor, N (%)	23 (33.0)	350 (60.4)	124 (68.1)	< 0.001
diet poor, N (%)	14 (13.6)	151 (26.1)	90 (49.5)	< 0.001
new antihypertensive medication at FU, N (%)	13 (12.6)	109 (18.8)	34 (18.7)	0.311
new anticholesterol medication at FU, N (%)	9 (8.7)	66 (11.4)	39 (21.4)	0.001
**Old-old (n = 158)**				
N (%)	18 (11.4)	107 (67.7)	33 (20.9)	
Age, M (SD)	79.9 (2.2)	80.5 (3.1)	81.4 (3.1)	0.162
Female, N (%)	10 (55.6)	71 (66.4)	19 (57.6)	0.507
Years of education, M (SD)	14.1 (3.9)	11.8 (4.0)	12.4 (3.9)	0.085
MMSE score, M (SD)	29.3 (0.8)	28.9 (1.0)	29.1 (0.9)	0.299
*APOE* E4 carrier, N (%)	3 (16.7)	29 (27.4)	8 (24.2)	0.619
CVD, N (%)	3 (16.7)	20 (18.7)	9 (27.3)	0.519
LS7, M (SD)	9.6 (1.5)	7.6 (1.5)	6.6 (1.5)	< 0.001
BMI poor, N (%)	1 (5.6)	14 (13.1)	8 (24.2)	0.2
smoking poor, N (%)	0 (0.0)	6 (5.6)	1 (3.0)	0.648
physical activity poor, N (%)	0 (0.0)	12 (11.2)	6 (18.2)	0.145
cholesterol poor, N (%)	5 (27.8)	41 (38.3)	20 (60.6)	0.019
glucose poor, N (%)	1 (5.6)	10 (9.3)	1 (3.0)	0.208
blood pressure poor, N (%)	5 (27.8)	86 (80.4)	29 (87.9)	< 0.001
diet poor, N (%)	1 (5.6)	19 (17.8)	15 (45.5)	0.004
new antihypertensive medication at FU, N (%)	6 (33.3)	25 (23.4)	7 (21.2)	0.6
new anticholesterol medication at FU, N (%)	2 (11.1)	18 (16.8)	9 (27.3)	0.28

All variables were assessed at baseline unless otherwise indicated. M = mean, SD = standard deviation, MMSE = Mini002DMental State Examination, *APOE =* Apolipoprotein E, CVD = cardiovascular disease, LS7 = Life’s Simple 7, BMI = body mass index, FU = follow-up

The proportion of those that improved their LS7 individual items was highest for cholesterol (31.1%), followed by diet (25.2%), and blood pressure (24.6%) (see Supplementary Table 2). The most pronounced deterioration was observed in glucose (24.7%) and physical activity (19.6%). Results from the multinomial logistic regression showed that individuals with poor or intermediate baseline LS7 scores were more likely to improve in their BMI, smoking, physical activity, diet and cholesterol scores over time, compared to the optimal group (*p* < 0.05). Similarly, the poor and intermediate groups were less likely to deteriorate in physical activity, diet, cholesterol, and blood pressure (*p* < 0.05).

Results from the linear-mixed models are presented in Table 3. Both worsening and improvement of LS7 total score were associated with faster decline in episodic memory (β = −0.02, *p* = 0.005 for worse and β = −0.02, *p* = 0.004 for improved), and global cognition (β = −0.01, *p* = 0.024 for worse and β = −0.01, *p* = 0.016 for improved) in the total sample. Reduced physical activity was associated with faster perceptual speed and global cognitive decline (see Supplementary Table 3).

**Table 3:** Estimates from linear mixed-effects models by LS7 group for the total sample, young-old, and old-old

Cognitive test	Worse			Improved		
	Beta	P value	95% CI	Beta	P value	95% CI
**Total Sample**						
episodic memory	−0.02*	0.005	−0.04, −0.01	−0.02*	0.004	−0.03, −0.01
semantic memory	−0.01	0.275	−0.02, 0.01	−0.01	0.333	−0.02, 0.01
verbal fluency	−0.01	0.014	−0.02, 0.00	−0.01	0.055	−0.02, 0.00
perceptual speed	0.00	0.964	−0.01, 0.01	0.00	0.535	−0.01, 0.01
global cognition	−0.01*	0.024	−0.02, 0.00	−0.01*	0.016	−0.02, 0.00
**Young-old**						
episodic memory	−0.02	0.088	−0.03, 0.00	−0.02	0.024	−0.03, 0.00
semantic memory	0.00	0.996	−0.01, 0.01	−0.01	0.332	−0.02, 0.01
verbal fluency	−0.01	0.153	−0.02, 0.00	−0.01	0.083	−0.02, 0.00
perceptual speed	0.01	0.052	0.00, 0.03	0.00	0.682	−0.01, 0.01
global cognition	0.00	0.590	−0.01, 0.01	−0.01*	0.025	−0.02, 0.00
**Old-old**						
episodic memory	−0.05*	0.002	−0.09, −0.02	−0.02	0.258	−0.04, 0.01
semantic memory	−0.05	0.046	−0.10, 0.00	0.00	0.967	−0.04, 0.04
verbal fluency	−0.05*	0.005	−0.08, −0.01	−0.01	0.419	−0.04, 0.02
perceptual speed	−0.09*	0.000	−0.13, −0.05	−0.01	0.688	−0.04, 0.02
global cognition	−0.06*	0.000	−0.08, −0.03	−0.01	0.423	−0.03, 0.01

Shown beta coefficients, confidence intervals, and p−values represent differences in rate of change compared to the stable LS7 group. Significant results are marked with an asterisk (*). A significance level of 0.0125 was applied for the specific domains of episodic memory, semantic memory, verbal fluency, and perceptual speed, while a value of 0.05 was used for global cognition. Analyses were controlled for age at baseline, sex, years of education, and baseline LS7

We found significant interactions between age and LS7 change with regard to cognitive decline and repeated the analyses in age-stratified groups. The effects of worse LS7 on cognitive decline were more pronounced in old-old individuals, where worsening of LS7 was associated with faster decline in episodic memory, verbal fluency, perceptual speed, and global cognition. In this age group, deteriorations in blood pressure, physical activity, and smoking were associated with global cognitive decline (see Table 4). Moreover, individuals who experienced deterioration in their blood pressure scores exhibited faster decline in episodic memory, while those who reduced their levels of physical activity experienced faster perceptual speed decline. There were no significant associations of LS7 improvements with rate of cognitive change in the old-old participants. In young-old individuals, those that improved their LS7 total score experienced faster decline in global cognition, although the effect size was small. No significant associations were observed for change in LS7 individual metrics in the young-old (see Supplementary Table 4).

**Table 4:** Estimates from linear mixed-effects models for LS7 individual metrics for old−old individuals

Cognitive test	Worse			Improved		
	Beta	P value	95% CI	Beta	P value	95% CI
**Smoking**						
episodic memory	−0.10	0.142	−0.24, 0.03	0.01	0.676	−0.05, 0.07
semantic memory	0.07	0.557	−0.17, 0.32	−0.02	0.723	−0.10, 0.07
verbal fluency	−0.16	0.016	−0.29, −0.03	0.00	0.882	−0.07, 0.06
perceptual speed	−0.19	0.014	−0.34, −0.04	0.00	0.947	−0.07, 0.08
global cognition	−0.14*	0.010	−0.24, −0.03	0.00	0.892	−0.05, 0.05
**Diet**						
episodic memory	−0.01	0.579	−0.04, 0.02	−0.01	0.644	−0.03, 0.02
semantic memory	−0.02	0.481	−0.07, 0.03	0.04	0.078	0.00, 0.08
verbal fluency	−0.01	0.455	−0.05, 0.02	−0.01	0.722	−0.03, 0.02
perceptual speed	0.01	0.764	−0.04, 0.05	0.01	0.711	−0.03, 0.04
global cognition	0.00	0.813	−0.03, 0.02	0.01	0.408	−0.01, 0.03
**Physical activity**						
episodic memory	−0.02	0.131	−0.05, 0.01	0.02	0.303	−0.02, 0.05
semantic memory	−0.04	0.044	−0.08, 0.00	−0.03	0.183	−0.08, 0.02
verbal fluency	−0.02	0.174	−0.05, 0.01	−0.01	0.417	−0.05, 0.02
perceptual speed	−0.05*	0.005	−0.08, −0.01	−0.02	0.234	−0.07, 0.02
global cognition	−0.03*	0.001	−0.06, −0.01	−0.01	0.310	−0.04, 0.01
**Body mass index**						
episodic memory	−0.03	0.180	−0.06, 0.01	−0.03	0.067	−0.06, 0.00
semantic memory	0.02	0.423	−0.03, 0.08	0.01	0.643	−0.04, 0.06
verbal fluency	0.01	0.791	−0.03, 0.05	−0.02	0.297	−0.05, 0.01
perceptual speed	−0.02	0.516	−0.06, 0.03	0.02	0.250	−0.02, 0.06
global cognition	0.00	0.821	−0.04, 0.03	0.00	0.788	−0.03, 0.02
**Blood glucose**						
episodic memory	0.00	0.705	−0.02, 0.03	−0.01	0.664	−0.05, 0.03
semantic memory	−0.02	0.410	−0.05, 0.02	−0.01	0.852	−0.06, 0.05
verbal fluency	0.02	0.104	0.00, 0.05	0.02	0.336	−0.02, 0.06
perceptual speed	0.00	0.985	−0.03, 0.03	0.01	0.606	−0.04, 0.06
global cognition	0.01	0.514	−0.01, 0.03	0.01	0.737	−0.03, 0.04
**Total cholesterol**						
episodic memory	0.00	0.955	−0.04, 0.04	−0.02	0.195	−0.04, 0.01
semantic memory	−0.01	0.766	−0.06, 0.05	−0.01	0.445	−0.05, 0.02
verbal fluency	−0.02	0.292	−0.06, 0.02	0.01	0.488	−0.02, 0.03
perceptual speed	−0.04	0.096	−0.09, 0.01	0.01	0.481	−0.02, 0.04
global cognition	−0.01	0.517	−0.04, 0.02	0.00	0.846	−0.02, 0.02
**Blood pressure**						
episodic memory	−0.05*	0.002	−0.08, −0.02	−0.01	0.524	−0.03, 0.02
semantic memory	−0.03	0.152	−0.08, 0.01	0.01	0.462	−0.02, 0.05
verbal fluency	−0.03	0.036	−0.07, 0.00	−0.01	0.455	−0.04, 0.02
perceptual speed	−0.04	0.032	−0.08, 0.00	0.00	0.987	−0.03, 0.03
global cognition	−0.04*	0.005	−0.06, −0.01	0.00	0.872	−0.02, 0.02

Shown beta coefficients, confidence intervals, and p−values represent differences in rate of change compared to the stable LS7 group. Significant results are marked with an asterisk (*). A significance level of 0.0125 was applied for the specific domains of episodic memory, semantic memory, verbal fluency, and perceptual speed, while a value of 0.05 was used for global cognition. Analyses were controlled for age at baseline, sex, years of education, and baseline LS7

## Discussion

In this prospective cohort study of individuals aged 60 years and older, we examined the association between changes in cardiovascular health over 6 years, measured with LS7, and rate of cognitive change over a span of 15 years. We found that a deterioration in LS7 score was associated with faster cognitive decline. Among individuals aged 78 years and older, a deterioration in LS7 was associated with faster decline in a variety of cognitive domains. These results were driven mostly by changes in blood pressure, physical activity, and smoking. In contrast, among younger participants, an improvement in LS7 tended to be associated with faster rate of cognitive decline.

Over a period of 6 years, the participants showed a slight improvement (M = 0.3, SD = 1.6) in their LS7 score, indicating relatively good overall health. Lassale et al. [29] studied individuals in midlife, and reported that 76% remained in their respective LS7 group after 6 years. Similarly, in our sample, 67% of participants were categorised as belonging to the stable LS7 group. On average, the greatest improvements were observed for cholesterol and diet, while the most notable declines were observed in glucose and physical activity levels. These results are consistent with a study on middle-aged individuals, where the greatest decline was observed in physical activity, followed by glucose levels, while smoking and cholesterol were the only metrics that showed improvement during the 10-year follow-up [30]. Different patterns were reported by Lassale et al., who found that older age was associated with improvements in smoking status, BMI, diet, and physical activity metrics, and lower odds of improvement in blood pressure.

### Total sample and old-old participants

Individuals with either poor or intermediate LS7 baseline scores, compared to the optimal group, were more likely to improve and less likely to deteriorate their LS7 score over time. This may partly be due to a floor effect preventing this category from declining further. Nevertheless, the effect of changes in cardiovascular health on cognitive decline was apparent even after controlling for LS7 at baseline. Individuals who experienced a deterioration of their LS7 score showed accelerated decline in episodic memory and global cognition. This is consistent with a finding from the Betula project, which showed that an accelerated cardiovascular risk trajectory over 20 to 25 years was associated with increased risk of episodic memory decline in healthy adults [13]. Another study found that reducing cardiovascular risk factors over a two-year period led to less decline in hippocampal volume [14], a variable that has been consistently linked to cognition, particularly episodic memory [31]. Similar to the Betula study, which only investigated the episodic memory domain, we found that worsening of cardiovascular health was associated with episodic memory, as well as verbal fluency, perceptual speed, and global cognitive decline among individuals aged 78 years and older, where the strongest association was observed for perceptual speed. Several underlying mechanisms have been proposed linking VRF to brain pathology, including white matter hyperintensities, which have been linked to perceptual speed, and hippocampal atrophy, which is associated with episodic memory [32].

Regarding individual metrics, significant associations were found for physical activity, where a decrease was associated with processing speed slowing and global cognitive decline in the total sample. Physical activity has been associated with increased blood flow, preservation of brain volume, and reduced risk of amyloid and tau pathology [33]. Noroozian et al. (2022) [34] reviewed the effect of cardiovascular risk factors on cognitive decline prevention in elderly, and found the most positive evidence for being physically active. In the old-old, deterioration of physical activity, together with blood pressure and smoking, was associated with faster cognitive decline. Hypertension can impact cognition through various mechanisms, including structural changes in blood vessels resulting in reduced blood flow to the brain, the development of white matter lesions, increased oxidative stress, and inflammation [35, 36]. Although only a few individuals started to smoke in this age group, a negative change in this metric was still associated with faster global cognitive decline, in line with previous findings of the association between smoking and cognitive decline in older adults [37]. Changes in these metrics may further reflect an overall worsening of physical health, which may impact cognitive functioning. Moreover, they may be associated with increased risk of cardiovascular events. In older adults, the development of new or additional vascular diagnoses has been linked to faster cognitive decline [38].

Interestingly, those who improved their LS7 score also experienced faster episodic memory and global cognitive decline in the total sample, while the improvement of LS7 did not show any significant effects on cognitive change in the old-old. This divergent pattern could be attributed to the age-dependent association between certain VRF and cognitive decline [3, 4]. In other words, improvement in LS7 factors such as cholesterol, BMI, and glucose may not be associated with improved cognition in older individuals. Rather, they may reflect negative health changes, such as impending dementia. Interventions studies targeting VRF in very old adults have shown diverse effects on cognition. Results from the FINGER project in Finland show that a multidomain intervention including diet, exercise, cognitive training, and vascular risk monitoring may improve or maintain cognitive functioning of non-demented elderly people aged 60–77 years with increased cardiovascular risk [39]. In contrast, a 3-year intervention including physical activity, cognitive training, nutritional advice, and omega 3 supplementation did not have significant effects on cognitive decline in individuals aged 70 years and older with memory complaints [40]. Similarly, in the Dutch Prevention of Dementia by Intensive Vascular Care (PreDIVA) trial, a multidomain intervention targeting cardiovascular risk factors in individuals aged 70–78 years did not result in a reduced incidence of all-cause dementia [41] However, in additional analyses, the intervention had a protective effect for non-Alzheimer dementia. Discrepancies in these outcomes may, at least in part, be attributed to variations in study designs. Multi-domain interventions targeting dementia risk factors remain a key approach in dementia prevention [42, 43], nevertheless, the existing studies in older adult population demonstrate the challenge in achieving consistent outcomes across diverse age groups and underscore the need for further research and tailored approaches in the field of dementia.

### Young-old participants

In young-old individuals, as in the total sample, improvement in LS7 score was linked to faster global cognitive decline. This somehow unexpected finding can be explained in several ways. In the improvement group, significantly more participants received new cholesterol medications during the 6-year follow-up, indicating struggles with their cholesterol health. The change in their LS7 may reflect their efforts to address their relatively poor baseline health status. It is also possible that improvements in cardiovascular health occurred too late in the progression of cognitive decline to effectively slow down the process. Even though participants improved their cardiovascular health, e.g. through medical treatment, cognitive decline may still continue to progress. Additionally, in old age, medication use does not always positively affect cognition; it may even have adverse cognitive effects [44, 45].

Another factor to consider is that the improvement group had poor or intermediate LS7 at baseline, which limits the range of LS7 score changes within this group. In other words, if individuals start with poor LS7, their health can only improve or remain stable, with no room for further deterioration. Individuals with poor baseline scores, compared to optimal, in cholesterol, diet, physical activity, smoking, and BMI were more likely to improve their LS7 score over time, supporting this notion. Moreover, it is important to note that an improvement in LS7 score may not always indicate a positive outcome. For example, a decrease in blood pressure and BMI levels could potentially be a sign of an impending dementia disorder [46, 47], suggesting that reverse causation may explain these findings. Possible reasons for why decline in LS7 score did not have a negative effect in the young-old are that young-old individuals are more resilient or that the changes may reflect minor or less severe health events compared to in the old-old.

### Strengths and limitations

To our knowledge, this is one of few studies examining the association between changes in multiple VRF and cognitive decline in different domains. Additional strengths of our study include a population-based sample, which was followed for many years, and a detailed assessment of multiple cognitive domains.

Some limitations should also be mentioned. Participants with poor scores on some biological metrics were advised to contact their physician for further investigation. Thus, this was not a purely observational study, but it did include certain feedback to the participants. Moreover, exclusion due to missing data and selective dropout likely contributed to an overestimation of the cardiovascular health in this population, as well as an underestimation of the effects of LS7 on rate of cognitive change.

### Implications

We found that worsening of LS7 was associated with faster rates of cognitive change, especially among older participants. It is noteworthy that although LS7 baseline scores significantly predicted change in LS7 over time, the effect of cardiovascular change on cognition was apparent even after controlling for LS7 baseline status. This underscores the importance of maintaining good cardiovascular health also in very old age and to, above all, avoid worsening of VRF status. Individuals who improved their LS7, especially young-old participants, also experienced faster global cognitive decline. These findings suggest that even a positive change may be associated with harmful effects on the cognitive status of older adults. This study constitutes an important extension to previous cross-sectional studies on associations between cardiovascular health and cognitive decline in old age. We have previously shown that having an optimal LS7 score was mainly important in young-old age in order to reduce the risk for cognitive decline. Here, we are able to demonstrate that maintaining a good cardiovascular health status is an important goal also in very old age. This study further highlights the complex relationships between cardiovascular health and cognition in old age, emphasizing the need for longitudinal studies to better understand the underlying mechanisms.

### Electronic supplementary material

Below is the link to the electronic supplementary material.

Supplementary Material 1

## Data Availability

The data that support the findings from this study are available from the SNAC-K database committee upon reasonable request. Applications for data use can be submitted by the following e-mail: maria.wahlberg@ki.se.
