# Supplementary material for: Change in cardiovascular health and rate of cognitive decline in older adults: a 15-year population-based study
Source: BMC Geriatr. 2024 Mar 18;24:263. doi: 10.1186/s12877-024-04856-y (PMC10949596; doi:10.1186/s12877-024-04856-y)
Supplement: Supplementary file 1 — Supplementary Material 1 [file 12877_2024_4856_MOESM1_ESM.docx]

Supplementary Table 1: Summary of variables measured in SNAC-K

| **Variable category** | **Description** |
| --- | --- |
| **Sociodemographic** | Age, sex, education |
|  | Occupational history |
|  | Marital status |
| **Social and physical environment** | Social network and support |
|  | Housing |
|  | Physical environment |
| **Self-reported health and well-being** | Sleep |
|  | Pain |
|  | Personality |
|  | Psychiatric symptoms |
|  | Quality of life |
| **Lifestyle and behaviors** | Smoking |
|  | Alcohol consumption |
|  | Diet |
|  | Physical activity |
|  | Leisure activities |
| **Disability and physical functioning** | Activities of daily living (ADL) |
|  | Instrumental ADL |
|  | Physical performance tests |
|  | Blood pressure |
|  | Height, weight |
| **Medical exam and clinical diagnoses** | Neurological exam |
|  | Health status |
|  | Current diseases and symptoms |
|  | Medical history |
|  | Medications |
| **Cognitive functioning** | Global cognitive functioning |
|  | Dementia screening |
|  | Neuropsychological test battery |
| **Informal and formal care** | Hospitalization |
|  | Visit to primary and specialists care |
|  | Home care |
|  | Care-giver/taker |
| **Biomarkers** | Electrocardiogram |
|  | Lab tests |
|  | Genotyping |
| **Medical diagnoses** | Information on health history from the Swedish National Patient Register |
| **Vital status** | Information on date and cause of death from the National Board of Health and Welfare |

Supplementary Table 2: Distribution of change in LS7 individual items

| LS7  individual  metrics | total sample (n = 1022) | | | | | | young-old (n = 864) | | | | | | old-old (n = 158) | | | | | |
| --- | --- | --- | --- | --- | --- | --- | --- | --- | --- | --- | --- | --- | --- | --- | --- | --- | --- | --- |
|  | worse |  | stable |  | improved |  | worse | | stable | | improved | | worse | | stable | | improved | |
|  | N | % | N | % | N | % | N | % | N | % | N | % | N | % | N | % | N | % |
| body mass index | 120 | 11.7 | 801 | 78.4 | 101 | 9.9 | 106 | 12.3 | 683 | 79.1 | 75 | 8.7 | 14 | 8.9 | 118 | 74.7 | 26 | 16.5 |
| smoking | 17 | 1.7 | 900 | 88.1 | 105 | 10.3 | 16 | 1.9 | 747 | 86.5 | 101 | 11.7 | 1 | 0.6 | 153 | 96.8 | 4 | 2.5 |
| physical activity | 200 | 19.6 | 608 | 59.5 | 214 | 20.9 | 157 | 18.2 | 512 | 59.3 | 195 | 22.6 | 43 | 27.2 | 96 | 60.8 | 19 | 12.0 |
| cholesterol | 110 | 10.8 | 594 | 58.1 | 318 | 31.1 | 97 | 11.2 | 508 | 58.8 | 259 | 30.0 | 13 | 8.2 | 86 | 54.4 | 59 | 37.3 |
| glucose | 252 | 24.7 | 719 | 70.4 | 51 | 5.0 | 209 | 24.2 | 616 | 71.3 | 39 | 4.5 | 43 | 27.2 | 103 | 65.2 | 12 | 7.6 |
| blood pressure | 166 | 16.2 | 605 | 59.2 | 251 | 24.6 | 146 | 16.9 | 507 | 58.7 | 211 | 24.4 | 20 | 12.7 | 98 | 62.0 | 40 | 25.3 |
| diet | 145 | 14.2 | 619 | 60.6 | 258 | 25.2 | 127 | 14.7 | 515 | 59.6 | 222 | 25.7 | 18 | 11.4 | 104 | 65.8 | 36 | 22.8 |

Supplementary Table 3: Estimates from linear mixed models for LS7 individual metrics for the total sample.

| Cognitive test | Worse | | | Improved | | |
| --- | --- | --- | --- | --- | --- | --- |
|  | Beta | P value | 95 % CI | Beta | P value | 95 % CI |
| **Smoking** | | | | | | |
| episodic memory | 0.01 | 0.531 | -0.03, 0.05 | -0.01 | 0.259 | -0.03, 0.01 |
| semantic memory | -0.01 | 0.497 | -0.05, 0.02 | 0.00 | 0.741 | -0.02, 0.01 |
| verbal fluency | 0.01 | 0.641 | -0.02, 0.03 | 0.00 | 0.718 | -0.01, 0.01 |
| perceptual speed | -0.02 | 0.231 | -0.05, 0.01 | 0.00 | 0.541 | -0.01, 0.02 |
| global cognition | -0.01 | 0.633 | -0.03, 0.02 | 0.00 | 0.797 | -0.01, 0.01 |
| **Diet** | | | | | | |
| episodic memory | -0.01 | 0.419 | -0.02, 0.01 | 0.00 | 0.537 | -0.01, 0.02 |
| semantic memory | 0.00 | 0.679 | -0.02, 0.01 | 0.01 | 0.260 | 0.00, 0.02 |
| verbal fluency | 0.00 | 0.884 | -0.01, 0.01 | 0.01 | 0.031 | 0.00, 0.02 |
| perceptual speed | 0.01 | 0.054 | 0.00, 0.02 | 0.01 | 0.109 | 0.00, 0.02 |
| global cognition | 0.00 | 0.641 | -0.01, 0.01 | 0.01 | 0.063 | 0.00, 0.01 |
| **Physical activity** | | | | | | |
| episodic memory | -0.01 | 0.271 | -0.02, 0.01 | 0.00 | 0.542 | -0.02, 0.01 |
| semantic memory | -0.01 | 0.090 | -0.02, 0.00 | 0.01 | 0.229 | 0.00, 0.02 |
| verbal fluency | -0.01 | 0.178 | -0.02, 0.00 | 0.01 | 0.134 | 0.00, 0.02 |
| perceptual speed | -0.01* | 0.009 | -0.03, 0.00 | 0.00 | 0.363 | -0.02, 0.01 |
| global cognition | -0.01* | 0.001 | -0.02, -0.01 | 0.00 | 0.956 | -0.01, 0.01 |
| **Body mass index** | | | | | | |
| episodic memory | 0.00 | 0.712 | -0.02, 0.01 | -0.01 | 0.459 | -0.03, 0.01 |
| semantic memory | 0.00 | 0.519 | -0.01, 0.02 | 0.00 | 0.551 | -0.02, 0.01 |
| verbal fluency | 0.01 | 0.024 | 0.00, 0.02 | -0.01 | 0.244 | -0.02, 0.00 |
| perceptual speed | 0.01 | 0.070 | 0.00, 0.03 | 0.00 | 0.566 | -0.02, 0.01 |
| global cognition | 0.01 | 0.174 | 0.00, 0.02 | -0.01 | 0.283 | -0.02, 0.00 |
| **Glucose** | | | | | | |
| episodic memory | 0.01 | 0.387 | -0.01, 0.02 | -0.01 | 0.540 | -0.03, 0.02 |
| semantic memory | 0.00 | 0.527 | -0.01, 0.01 | -0.01 | 0.261 | -0.03, 0.01 |
| verbal fluency | 0.00 | 0.314 | 0.00, 0.01 | -0.01 | 0.095 | -0.03, 0.00 |
| perceptual speed | 0.00 | 0.857 | -0.01, 0.01 | -0.01 | 0.416 | -0.03, 0.01 |
| global cognition | 0.00 | 0.378 | 0.00, 0.01 | -0.01 | 0.133 | -0.02, 0.00 |
| **Cholesterol** | | | | | | |
| episodic memory | 0.01 | 0.273 | -0.01, 0.03 | 0.00 | 0.838 | -0.01, 0.01 |
| semantic memory | 0.00 | 0.901 | -0.02, 0.01 | -0.01 | 0.154 | -0.02, 0.00 |
| verbal fluency | 0.00 | 0.912 | -0.01, 0.01 | 0.00 | 0.817 | -0.01, 0.01 |
| perceptual speed | 0.01 | 0.465 | -0.01, 0.02 | 0.00 | 0.337 | -0.01, 0.00 |
| global cognition | 0.00 | 0.405 | -0.01, 0.01 | 0.00 | 0.341 | -0.01, 0.00 |
| **Blood pressure** | | | | | | |
| episodic memory | -0.01 | 0.068 | -0.03, 0.00 | 0.00 | 0.481 | -0.02, 0.01 |
| semantic memory | 0.00 | 0.785 | -0.01, 0.01 | 0.00 | 0.964 | -0.01, 0.01 |
| verbal fluency | 0.00 | 0.835 | -0.01, 0.01 | 0.00 | 0.259 | -0.01, 0.00 |
| perceptual speed | 0.00 | 0.721 | -0.01, 0.01 | 0.00 | 0.463 | -0.01, 0.01 |
| global cognition | 0.00 | 0.485 | -0.01, 0.01 | 0.00 | 0.372 | -0.01, 0.00 |

Shown beta coefficients, confidence intervals, and p-values represent differences in rate of change compared to the stable LS7 group. Significant results are marked with an asterisk (*). A significance level of 0.0125 was applied for the specific domains of episodic memory, semantic memory, verbal fluency, and perceptual speed, while a value of 0.05 was used for global cognition. Analyses were controlled for age at baseline, sex, years of education, and baseline LS7.

Supplementary Table 4: Estimates from linear mixed models for LS7 individual metrics for young-old individuals.

| Cognitive test | Worse | | | Improved | | |
| --- | --- | --- | --- | --- | --- | --- |
|  | Beta | P value | 95 % CI | Beta | P value | 95 % CI |
| **Smoking** | | | | | | |
| episodic memory | 0.02 | 0.302 | -0.02, 0.06 | -0.02 | 0.080 | -0.03, 0.00 |
| semantic memory | -0.02 | 0.192 | -0.05, 0.01 | -0.01 | 0.326 | -0.02, 0.01 |
| verbal fluency | 0.01 | 0.399 | -0.01, 0.04 | 0.00 | 0.692 | -0.01, 0.01 |
| perceptual speed | -0.01 | 0.379 | -0.04, 0.02 | 0.00 | 0.959 | -0.01, 0.01 |
| global cognition | 0.00 | 0.917 | -0.02, 0.02 | -0.01 | 0.172 | -0.02, 0.00 |
| **Diet** | | | | | | |
| episodic memory | 0.00 | 0.913 | -0.02, 0.02 | 0.01 | 0.458 | -0.01, 0.02 |
| semantic memory | 0.00 | 0.862 | -0.01, 0.01 | 0.00 | 0.870 | -0.01, 0.01 |
| verbal fluency | 0.00 | 0.983 | -0.01, 0.01 | 0.01 | 0.021 | 0.00, 0.02 |
| perceptual speed | 0.01 | 0.042 | 0.00, 0.03 | 0.01 | 0.124 | 0.00, 0.02 |
| global cognition | 0.00 | 0.348 | 0.00, 0.01 | 0.01 | 0.093 | 0.00, 0.01 |
| **Physical activity** | | | | | | |
| episodic memory | 0.00 | 0.642 | -0.01, 0.02 | 0.00 | 0.785 | -0.02, 0.01 |
| semantic memory | 0.00 | 0.631 | -0.01, 0.01 | 0.01 | 0.092 | 0.00, 0.02 |
| verbal fluency | 0.00 | 0.594 | -0.01, 0.01 | 0.01 | 0.144 | 0.00, 0.02 |
| perceptual speed | -0.01 | 0.188 | -0.02, 0.00 | -0.01 | 0.344 | -0.02, 0.01 |
| global cognition | 0.00 | 0.235 | -0.01, 0.00 | 0.00 | 0.775 | -0.01, 0.01 |
| **BMI** | | | | | | |
| episodic memory | 0.00 | 0.975 | -0.02, 0.02 | 0.00 | 0.932 | -0.02, 0.02 |
| semantic memory | 0.00 | 0.821 | -0.01, 0.02 | 0.00 | 0.712 | -0.02, 0.01 |
| verbal fluency | 0.01 | 0.030 | 0.00, 0.02 | 0.00 | 0.997 | -0.01, 0.01 |
| perceptual speed | 0.02 | 0.025 | 0.00, 0.03 | -0.01 | 0.423 | -0.02, 0.01 |
| global cognition | 0.01 | 0.143 | 0.00, 0.02 | 0.00 | 0.571 | -0.01, 0.01 |
| **Glucose** | | | | | | |
| episodic memory | 0.01 | 0.220 | 0.00, 0.02 | 0.00 | 0.933 | -0.03, 0.03 |
| semantic memory | 0.00 | 0.821 | -0.01, 0.01 | -0.01 | 0.614 | -0.03, 0.02 |
| verbal fluency | 0.00 | 0.447 | -0.01, 0.01 | -0.02 | 0.060 | -0.03, 0.00 |
| perceptual speed | 0.00 | 0.642 | -0.01, 0.01 | -0.01 | 0.494 | -0.03, 0.01 |
| global cognition | 0.00 | 0.230 | 0.00, 0.01 | -0.01 | 0.249 | -0.02, 0.01 |
| **Cholesterol** | | | | | | |
| episodic memory | 0.01 | 0.352 | -0.01, 0.03 | 0.00 | 0.531 | -0.01, 0.02 |
| semantic memory | 0.00 | 0.923 | -0.01, 0.01 | 0.00 | 0.439 | -0.01, 0.01 |
| verbal fluency | 0.00 | 0.663 | -0.01, 0.01 | 0.00 | 0.935 | -0.01, 0.01 |
| perceptual speed | 0.01 | 0.107 | 0.00, 0.03 | -0.01 | 0.300 | -0.01, 0.00 |
| global cognition | 0.01 | 0.172 | 0.00, 0.02 | 0.00 | 0.620 | -0.01, 0.00 |
| **Blood pressure** | | | | | | |
| episodic memory | -0.01 | 0.10 | -0.03, 0.00 | 0.00 | 0.658 | -0.02, 0.01 |
| semantic memory | 0.00 | 0.412 | -0.01, 0.02 | 0.00 | 0.705 | -0.01, 0.01 |
| verbal fluency | 0.00 | 0.721 | -0.01, 0.01 | 0.00 | 0.327 | -0.01, 0.00 |
| perceptual speed | 0.00 | 0.824 | -0.01, 0.01 | 0.00 | 0.405 | -0.01, 0.01 |
| global cognition | 0.00 | 0.892 | -0.01, 0.01 | 0.00 | 0.353 | -0.01, 0.00 |

Shown beta coefficients, confidence intervals, and p-values represent differences in rate of change compared to the stable LS7 group. Significant results are marked with an asterisk (*). A significance level of 0.0125 was applied for the specific domains of episodic memory, semantic memory, verbal fluency, and perceptual speed, while a value of 0.05 was used for global cognition. Analyses were controlled for age at baseline, sex, years of education, and baseline LS7.
